# Cognitive Flexibility Training: A Large-Scale Multimodal Adaptive Active-Control Intervention Study in Healthy Older Adults

**DOI:** 10.3389/fnhum.2017.00529

**Published:** 2017-11-01

**Authors:** Jessika I. V. Buitenweg, Renate M. van de Ven, Sam Prinssen, Jaap M. J. Murre, K. Richard Ridderinkhof

**Affiliations:** ^1^Department of Psychology, University of Amsterdam, Amsterdam, Netherlands; ^2^Amsterdam Brain and Cognition, University of Amsterdam, Amsterdam, Netherlands

**Keywords:** aging, cognitive training, executive functions, cognitive flexibility, videogames

## Abstract

As aging is associated with cognitive decline, particularly in the executive functions, it is essential to effectively improve cognition in older adults. Online cognitive training is currently a popular, though controversial method. Although some changes seem possible in older adults through training, far transfer, and longitudinal maintenance are rarely seen. Based on previous literature we created a unique, state-of-the-art intervention study by incorporating frequent sessions and flexible, novel, adaptive training tasks, along with an active control group. We created a program called TAPASS (Training Project Amsterdam Seniors and Stroke), a randomized controlled trial. Healthy older adults (60–80 y.o.) were assigned to a frequent- (FS) or infrequent switching (IS) experimental condition or to the active control group and performed 58 half-hour sessions over the course of 12 weeks. Effects on executive functioning, processing- and psychomotor speed, planning, verbal long term memory, verbal fluency, and reasoning were measured on four time points before, during and after the training. Additionally, we examined the explorative question which individual aspects added to training benefit. Besides improvements on the training, we found significant time effects on multiple transfer tasks in all three groups that likely reflected retest effects. No training-specific improvements were detected, and we did not find evidence of additional benefits of individual characteristics. Judging from these results, the therapeutic value of using commercially available training games to train the aging brain is modest, though any apparent effects should be ascribed more to expectancy and motivation than to the elements in our training protocol. Our results emphasize the importance of using parallel tests as outcome measures for transfer and including both active and passive control conditions. Further investigation into different training methods is advised, including stimulating social interaction and the use of more variable, novel, group-based yet individual-adjusted exercises.

## Introduction

We live in a time of great societal changes in the Western world. Due in part to dramatic improvements in medical science, our aging population is expanding rapidly. As aging is associated with decreased cognitive functioning, the prevalence of age-related cognitive decline is an increasingly important issue. Decline of cognitive control, memory, and decision-making, among other functions, leads to greater dependence on family members and society. With recent increments of the retirement age in many countries, increasing numbers of older workers are expected to contribute to the workforce, but may cognitively fall behind. In order to ensure that older adults can live and work independently for as long as possible, research into possibilities of reducing this age-related decline of functioning is a pressing matter.

Enhancing cognitive functions or limiting their decline using cognitive training is currently a popular topic. Effectiveness of such trainings has been investigated with numerous intervention studies, for instance working memory training (Buschkuehl et al., 2008; Richmond et al., 2011; Rose et al., 2015), virtual reality training (Optale et al., 2010; Lövdén et al., 2012), and game training (Basak et al., 2008; Nouchi et al., 2012; van Muijden et al., 2012; Baniqued et al., 2013; Toril et al., 2016). Benefits of using at-home computer-based training programs are evident: they require no face-to-face contact, are easy to administer, and do not require traveling, which is especially advantageous when catering to more physically impaired individuals. Furthermore, they are cost efficient, and can be customized to a personal level in order to keep motivation optimal. In addition to the possible benefits for cognition, young and older adults also enjoy playing computer games in order to challenge themselves and for reasons of entertainment and—for certain games—social rewards (Allaire et al., 2013; Whitbourne et al., 2013). The gaming industry has conveniently caught on to this trend. As a result, countless commercial training websites and stand-alone applications offer a whole range of games that promise to contribute to cognitive reserve and slowed decline.

Research indicates, however, that not all types of games are enjoyed equally by the older population. Realistic first-person shooter games, though cognitively challenging, are perceived negatively by many older adults (Nap et al., 2009; McKay and Maki, 2010). Generally, casual games or games comprised of short mathematical- or memory activities are rated as most enjoyable and lead to higher compliance and beliefs about enhancement (Nap et al., 2009; Boot et al., 2013a).

Despite its popularity and market potential, the effectiveness of brain training remains a controversial topic. Results are inconsistent (Au et al., 2015; Dougherty et al., 2016) with some producing no transfer effects at all (Ackerman et al., 2010; Lee et al., 2012). Near transfer is often reported, especially after multitasking or task-switching designs (Karbach and Kray, 2009; Wang et al., 2011; Anguera et al., 2013) though far transfer is scarcely found (Green and Bavelier, 2008; Park and Bischof, 2013). Furthermore, a large variability in the degree of individual response to cognitive training is often observed (Langbaum et al., 2009; Melby-Lervag et al., 2016). For instance, general training benefit is often found to be dependent on higher age and lower baseline cognitive abilities, and in some cases on training gain and education (Verhaeghen et al., 1992; Bissig and Lustig, 2007; Langbaum et al., 2009; Zinke et al., 2014) although there is some evidence of increasing benefit after lower baseline scores (Ball et al., 2007; Whitlock et al., 2012).

We and others (e.g., Buitenweg et al., 2012; Slagter, 2012; van de Ven et al., 2016) raised a number of problematic issues often encountered in the training research literature. Among them were brief training periods (limited numbers of session/days/weeks), small sample sizes, absence of active control conditions, inapt competitive motivational incentives, and use of unimodal training tasks (incurring task-specific and even stimulus-specific rather than process-specific training benefits). On the basis of our review of optimal study design, training efficacy, and neurocognitive profiles of successful aging (Buitenweg et al., 2012), we suggested adding the elements of flexibility, novelty (Noice and Noice, 2008) and adaptiveness (Kelly et al., 2014) to training protocols to increase the chances of finding positive effects on cognitive functioning.

Due to the encountered issues in the literature, the current situation in the training field is inconclusive on training generalizability. We therefore created a unique, state-of-the-art, 12-week intervention study incorporating multimodal, novel, adaptive training games and frequent sessions. To induce flexibility, we transformed the idea of task switching training, which has lead to far transfer in Karbach and Kray (2009). We integrated switching between training games to create a more ecologically valid intervention, while using a number of switching tasks as our transfer measures. Besides this, we employed a number of measures with alternate (parallel) forms in order to minimize retest effects. In addition to including a number of essential elements, we are the first study adding flexibility as a key ingredient to training. We were especially interested in the question whether shifting attention between multiple functions during the training would transfer to decreasing switch costs. For this purpose, we required a task in which to present both alternating and repeating cues, which was possible using the switching paradigm previously used by Rogers and Monsell (1995). However, to evaluate effects on the entire construct of task switching, we combined additional measures in our secondary analysis. We included the clinically validated Delis-Kaplan Executive Function System—Trail Making Test and the online version of this task, in which (unlike in our main switch task) every response requires a switch, but participants still access basic knowledge of number- and letter systems. To incorporate a more ecologically valid measure of task switching, we also added the switch condition of the semantic fluency test, in which switching between activations of more covert representations is required.

We investigated whether an online training incorporating these crucial components can lead to transfer in an elderly population. Our training program consisted of two experimental conditions and an active control condition in a program called TAPASS (Training Project Amsterdam Seniors and Stroke). The TAPASS program has been used to determine the effects of cognitive flexibility training in stroke survivors by adding to the usual rehabilitation care (van de Ven et al., 2015). Here, we focus on effectiveness of this program in the healthy aging population. Experimental groups differed in flexibility, novelty, and adaptiveness. Higher flexibility was created by having the subject switch more often in a session between cognitive domains from game to game. High novelty implied exposure to more different cognitive domains within one session. Adaptiveness refers to the extent to which game difficulty can be adapted dynamically to an individual's performance. The frequent switching (FS) group scored high on flexibility, novelty, and adaptiveness. The infrequent switching (IS) group contained high novelty and adaptiveness but low flexibility, and the mock training (MT) scored low on all three features. We investigated whether there are benefits of the experimental training on cognitive functioning, and if so, whether the switching component adds extra value to these effects. In addition, we explored the question whether training efficacy is modulated by individual characteristics, such as age, baseline functioning, or education.

As the current intervention is especially focused on inducing flexibility, we expected transfer to occur in functions of executive control (Buchler et al., 2008; Karbach and Kray, 2009; Buitenweg et al., 2012). Based on a classification model by Miyake et al. (2000) these are often separated into updating, inhibition, and shifting (dual tasking and task switching). Therefore, our main analysis was centered around measures of these constructs. For reasons of equal comparison, for the primary analysis we selected tasks that were all administered by computer at the lab.

In our secondary analysis, we included additional assessments of working memory and task switching as well as tasks from other domains. Due to their dependence on the frontal lobe, planning and verbal fluency are often counted among the executive functions as well (Fisk and Sharp, 2004; Phillips et al., 2006; Lewis and Miller, 2007) and can be subject to decline in older adults (Auriacombe et al., 2001; Sullivan et al., 2009; Kim et al., 2013). For this reason, we chose to include measures within these domains. In addition to the executive functions, processing speed often declines in later life (Salthouse, 2000), though training with a similar intervention has been seen to lead to improvements in this domain (Nouchi et al., 2012). As most of our training tasks included fast paced, timed games, we were interested to see whether the training would generalize to measures of processing speed. Additionally, as using the computer mouse was an important part in this study in completing the training tasks as well as the transfer measures, we also decided to include tasks of psychomotor speed. Finally, two more functions often found to diminish in older adults are reasoning ability and verbal long term memory (Davis et al., 2003; Harada et al., 2013), which have also seen improvements after similar interventions (Au et al., 2015; Barban et al., 2015). Measures of these constructs have been added to our battery of transfer tasks.

The purpose of this study was to test the hypothesis that a 12-week cognitive flexibility training would improve cognitive functions in healthy older adults. We expected to see the largest transfer effects on executive control performance after the frequent switch training, smaller effects after the infrequent switch training, and little to no effects after the MT. We expected differences between conditions to be smaller, yet in the same direction, on performance of other domains.

## Methods

### Subjects

Our study entailed a randomized controlled double-blind design. Participants were recruited via media campaigns (pitch talks on regional radio stations and articles in local newspapers) and from a database of healthy older adults interested in participation in psychological research (www.seniorlab.nl). A total of 249 healthy participants signed up online on www.tapass.nl and were assessed for eligibility. Inclusion criteria included age above 60, willingness and cognitive ability to finish the 12-week training program, and daily access to a computer with internet connection. Exclusion criteria were a history of neuropsychiatric disorders, TIA or stroke, strongly impairing visual deficits, and colorblindness. Additionally, mental condition was estimated with the Telephone Interview Cognitive Status (TICS; Brandt et al., 1988): individuals scoring below 26 on this test were excluded. Eleven individuals did not fit the inclusion criteria and were excluded. Twenty-nine individuals withdrew before randomization, and another 51 before the first test session, due to health- and technical issues, or lack of time. The remaining 158 subjects were included in the final sample.

**Figure 1:**
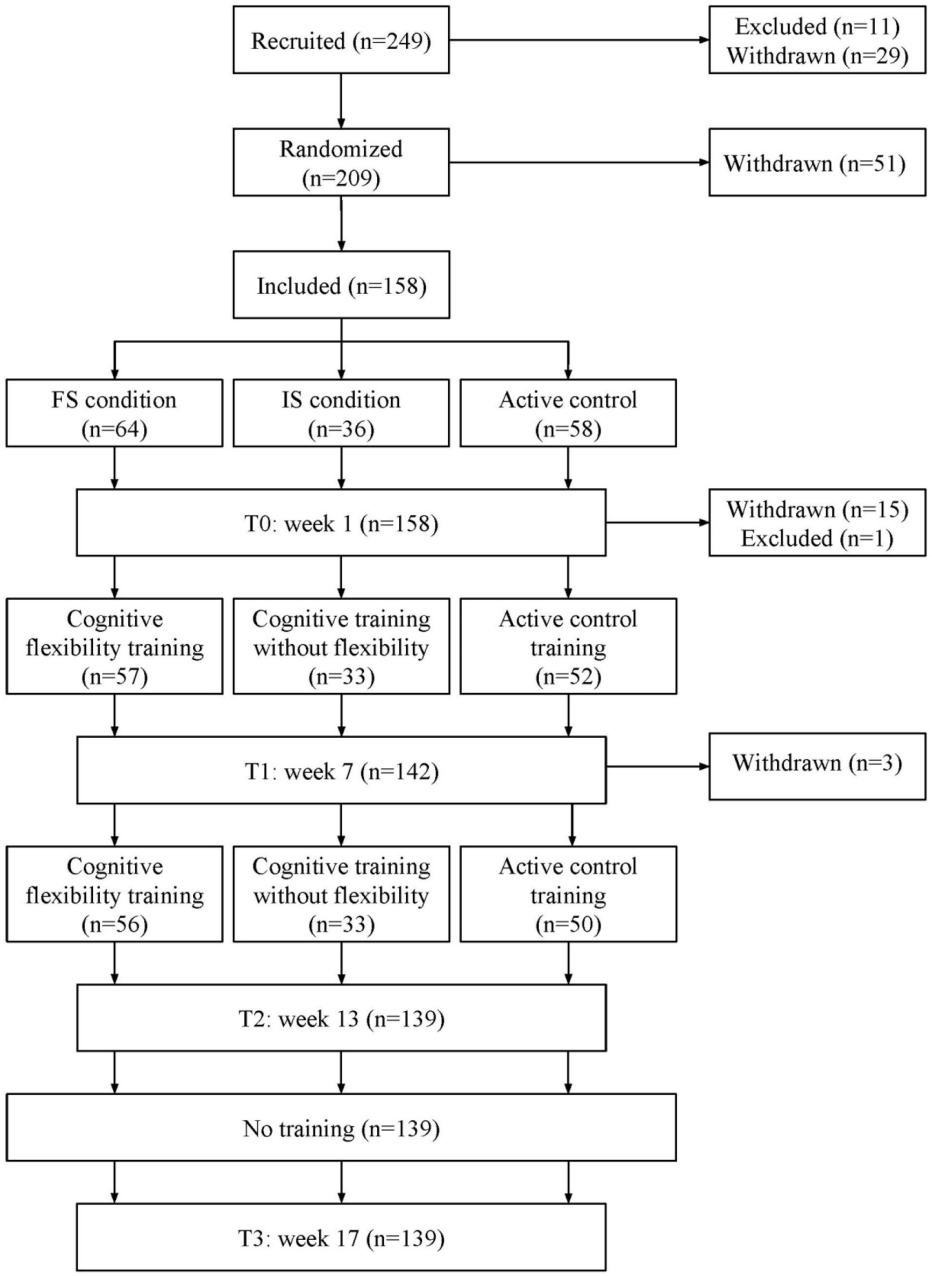
Flow chart of the study design. FS, frequent switching; IS, infrequent switching.

Subjects were randomly assigned to one of three conditions, with the exception of partners/spouses, who were always assigned to the same group. We minimized asymmetry in our three conditions using a minimization program (Minimpy; Saghaei and Saghaei, 2011) over the factors age, computer experience, TICS score, gender, and education. The minimization procedure was carried out by the principal investigators only. All subjects were given the same information regarding the intention of the experiment. They were told they would be placed in one of three different conditions, without explicit mention of a control condition. A schematic overview of the study design can be found in Figure 1.

Participants were compensated for travel costs and received free unlimited access to all games on www.braingymmer.com. Full written informed consent was given by all subjects prior to participation. The study was approved by the local Ethics Committee of the University of Amsterdam and registered under number 2012-BC-2566. All procedures were conducted in compliance with the Declaration of Helsinki, relevant laws, and institutional guidelines.

### Study protocol

A battery of online tests (Neurotask BV, 2012) was devised to measure effects of the training at four points in time: at baseline (T0), after 6 weeks of training (T1), after 12 weeks of training (T2), and 4 weeks post-training (T3). On T0 and T2, subjects also visited the university for a series of neuropsychological tests and computer tasks, and a small set of cognitive tests was administered via a link in the email. Additionally, subjective effects were measured using a series of questionnaires at all time points, and a subgroup of participants underwent Magnetic Resonance Imaging (MRI) scanning at T0 and T2. Results of these subjective and MRI measures will be reported separately. Testing on T0 and T2 was spread out over three different days, and on T1 and T3 over 2 days. Both the order of the test days for T0 and T2 and order of testing within the neuropsychological test battery were counterbalanced between subjects.

Neuropsychological assessments were conducted by a trained junior psychologist, who was blind to the training condition. As a check, neuropsychological assessors were asked to guess the condition of the subject. A separate test assessor administered four computer tasks, and introduced the training to subjects using instruction videos and a demonstration of the training platform and games. After their first visit to the university, subjects received a personalized instruction booklet with illustrations reminding them how to log on to the testing and training platforms, how to play each game, and how to report technical problems. It also gave useful information beyond the training, for example, how to download a new browser, and the importance of good posture during computer use. Subjects were assigned to a member of the research team who called them weekly to biweekly with standardized questions, would offer motivation and feedback and who could solve (technical) problems. Subjects were encouraged to email or call their contact with more urgent problems.

Subjects were requested to train five times a week for a half hour, on days and times of their choosing. Training activity was monitored. If no login was encountered for 2 days, an automatic email was sent to the subject. Subjects were encouraged to finish the training in 12 consecutive weeks. If training had to be interrupted for a period of more than 2 days, such as during a holiday, the missed trainings were added to the end of the 12 weeks.

After T3, participants filled out an exit questionnaire in which they were asked to rate the training and their own motivation. To verify blindness, we asked subjects to guess in which condition they had been included, in the case that one condition was less effective than the other. Subsequently, all subjects received login information for a lifetime account on the training website.

### Intervention

All three training programs were based on the brain training website www.braingymmer.com. Games were originally programmed for the general population, but after running a pilot study, we altered the ones selected for our programs to fit the need of older participants. For example, many games commenced at too high speed and difficulty levels. This was adjusted in the research-dedicated “dashboard” version of the platform. In this platform, game presentation order was fully preprogrammed in order to prevent individuals from selecting their own tasks. Subjects had some extra time to finish after the time-period set aside for playing a certain game had been reached (e.g., 3 or 10 min) to prevent too abruptly ending a game.

All groups received the same amount and type of feedback after finishing a game or training session (see Supplementary Material 1). Additionally, all participants received standardized weekly to bi-weekly feedback and support from research team members who supervised them from baseline until 4 weeks post-training.

### Cognitive training

We designed a cognitive training based on nine games in three domains: reasoning, working memory, and attention (see Supplementary Material 2). In designing our intervention, we chose not to include training games which too closely resembled any of our transfer tasks. Each game consisted of 20 levels, increasing in difficulty. The order of games was selected in such a way that two games following one another were never from the same domain, to optimize variability and flexibility.

Subject performance was rated with up to three stars at the end of each game block. Adaptiveness was implemented by asking subjects to continue to the next difficulty level when reaching two or three stars. In case a subject reached the highest level (20), he or she was asked to improve performance on previous levels with two stars.

Within the cognitive training we created two groups: frequent switching (FS) and infrequent switching (IS). In the FS group, one training session consisted of 10 games of 3 min each, thus requiring subjects to frequently switch to a task aimed to train a different cognitive function than the one before. In the IS group, three games of 10 min each were played so that switching between game domains occurred less frequently. In the first week only, in order for subjects to become familiar with the games, both groups played the games for 10 min each. By the end of the intervention, the time spent on each game was similar across participants in the FS and IS groups.

### Mock training

For the MT, we selected games that provided equal visual stimulation and feedback and put equal demands on computer ability, but that were reduced in variability, flexibility, and adaptiveness, compared with the experimental conditions (see Supplementary Material 3). We selected four games that all put minimal demands on executive functions. Per session, subjects played three games of 10 min each, thus minimizing the need for flexibility. Unlike the FS and IS conditions, the MT was not adaptive. Although higher levels could be unlocked in the same manner, participants in the MT were instructed to remain on the same level for a week before continuing to the next level, regardless of the number of stars they received on a game.

### Assessment tasks

The effects of the flexibility training were estimated using pre- and post-measures on an extensive battery of computer tasks, neuropsychological paper-and-pen tests and computerized versions of these tests. For detailed task descriptions, see van de Ven et al. (2015).

### Principal analysis

For the principal analysis we used the executive functions as distinguished by Miyake et al. (2000): shifting (task switching and dual tasking), updating, and response inhibition. These were assessed with four computerized tasks. Task switching and dual tasking performances were measured using modified versions of a commonly used switch task (Rogers and Monsell, 1995) and dual task (Stablum et al., 2007). The two tasks were combined to save time. Switch cost was calculated as the difference between reaction time on switch trials and no-switch trials in milliseconds, with higher switch cost signifying lower cognitive flexibility (Rogers and Monsell, 1995). Dual task performance was assessed by the reaction time on speeded responses of the dual trials (Stablum et al., 2007). Updating performance was measured using the N-back task as used by de Vries and Geurts (2014) including 0-back, 1-back, and 2-back blocks. Performance on this task was calculated by the difference between the percentage correct on 2-back and percentage correct on 0-back items (Kirchner, 1958). The stop-signal task (Logan et al., 1984) was used to measure inhibition. Stop-signal reaction time (SSRT) was calculated by sorting all correct Go-trial reaction times, taking the time corresponding to the percentage of correct stop trials, and subtracting the mean stop-signal delay (SSD) from this number (Logan et al., 1984).

### Secondary analysis

Effectiveness of the training on a larger scale was assessed by using neuropsychological tests from eight cognitive domains: task switching, psychomotor speed, processing speed, planning, reasoning, working memory, long term memory, and verbal fluency. In most domains we included multiple tests. For the RAVLT, letter fluency, category fluency with- and without switch condition, and Raven Progressive Matrices, we used alternate assessment forms. Where necessary, raw scores were recoded such that higher scores always represent better performance.

In the domain of task switching, we included the Delis-Kaplan Executive Function System—Trail Making Test (D-KEFS TMT; Condition 4), the Trail Making Test-B (TMT-B), and a separate switch condition of the semantic fluency task. The D-KEFS TMT concerned the number-letter switching subtask, with the performance score calculated as the total time in seconds to complete connecting letters and numbers in alternating order (i.e., 1, A, 2, B, etc.; (Delis et al., 2001). The TMT-B pertained to the online version of this task, with performance assessed by the total time in seconds to complete connecting letters and numbers in alternating order (NeuroTask BV). The switch condition of the semantic fluency task consisted of alternating listing as many words as possible from two separate categories (male names and supermarket items, or female names and cities, counterbalanced over participants) in 1 min. Outcome measure is the number of correct words in the switch condition, subtracted from the average number of correct words produced in the same categories without switching (Troyer et al., 1997).

For psychomotor speed, we used five tasks, four of which were assessed online. In the drag-and-drop task, participants were required to use their computer mouse to drag round or square shapes into an empty border. Outcome measure is the total time in milliseconds to complete the task (Neurotask BV). In the drag-to-grid task, participants dragged 25 squares into a 5 × 5 grid using the mouse. Performance was assessed by the total time in milliseconds to complete the task (Neurotask BV). The click task required participants to click a spiral of circles of decreasing sizes using the mouse, with total time in milliseconds to complete the task signifying the outcome measure (Neurotask BV). The D-KEFS TMT (Condition 5) concerned the motor speed condition, with the performance score calculated as the total time in seconds to complete tracing a dotted line between a number of circles (Delis et al., 2001). The TMT-A pertained to the online version of this task, with performance assessed by the total time in seconds to complete connecting numbers (NeuroTask BV).

Processing speed was measured using the Digit Symbol Coding test (DSC; Wechsler, 2000) and an online version of this task (Neurotask BV). In this task, participants are required to pair a series of numbers to the correct symbol according to a given rule. Outcome measure on this task is the correct number of items completed in 2 min.

In planning, we used the Tower of London (ToL). This concerned the online version (Neurotask BV) based on the original task by Culbertson and Zillmer (2005), in which participants move colored beads from a starting position into the required position using a minimum amount of possible steps. Performance was assessed by the sum of the number of additional moves to solve the ToL, using a maximum score of 20.

In the reasoning domain, we included Raven's Progressive Matrices (Raven et al., 1998) as well as the Shipley Institute of living scale-2 (Zachary, 1991). For both reasoning tasks, the outcome measure we used was the total number correct on 20 items.

Working memory was assessed using two online tasks and three face-to-face tasks. A modified version of the Corsi block tapping task (Milner, 1971) was constructed for online assessment. Outcome measure was the longest correctly reproduced array of blocks (Neurotask BV). In the Paced Auditory Serial Addition Task (PASAT), participants needed to update the addition of numbers presented auditively. We administered two versions, in which numbers were delivered at a rate of, respectively, 3.4 and 2.8 s. As an outcome measure, we calculated the mean percentage correct of both versions (Gronwall, 1977). The Operation Span consisted of a series of letters presented sequentially that needed to be remembered while solving mathematical equations (Unsworth et al., 2005). Outcome measure for the Operation Span was the total number of correctly remembered letters. In Rey's Auditory Verbal Learning Test (RAVLT)–direct, participants were presented with a series of words auditively for five trials and recalled as many words as possible after each trial. We used the total number of words remembered after five trials as an outcome measure (Saan and Deelman, 1986). Lastly, in Letter Number Sequencing (LNS), participants were required to recall a series of numbers and letters in increasing or alphabetical order (Wechsler, 2000). For this measure, we used the total number of correct items.

In verbal long term memory, the delayed item of the RAVLT was used, in which the outcome measure was the total number of words recalled after a delay of 20 min (Saan and Deelman, 1986).

In the domain of verbal fluency, we used a semantic fluency task and a letter fluency task. In the semantic fluency task, participants produced as many words as possible in two different categories (male names and supermarket items, or female names and cities, counterbalanced over participants), each in 1 min (Thurstone, 1938). In the letter fluency task, participants produced as many words as possible starting with one of three different letters (P, G, and R on one time point, K, O, and M on the other time point, counterbalanced over participants), each in 1 min (Benton et al., 1989). For both tests, the outcome measure was the mean number of correct words.

To control for possible differences in fatigue and depression, we also examined baseline scores of the Checklist Individual Strength—Fatigue subscale (CIS-F) and the Hospital Anxiety Depression Scale—Depression subscale (HADS-D). The HADS-D (Zigmond and Snaith, 1983) measures subjective severity of depression and includes seven items on a four-point scale, with a maximum score of 21. The CIS-F (Vercoulen et al., 1997) measures subjective fatigue and the behavioral characteristics related to this concept. The scale consists of eight items, with scores ranging from 8 to 56. A score of 35 is regularly used as a cut-off to denote severe fatigue (Worm-Smeitink et al., 2017).

We designed an exit scale with four separate questions assessing perceived difficulty and enjoyment of the games, self-rated general cognitive enhancement, and whether participants would continue using the training. Although the scale is not validated, it serves as a necessary tool to judge participants' present and future view of the training. Participants rated these questions on T2 and T3, on a five-point Likert scale.

### Training performance

Training performance in all three groups was measured using a mean training z-score as well as a mean gain score between T0 and T2. Level high scores were calculated as a percentage of the maximal score on that level. Next, all were added up to a total game score for each training game. For the experimental conditions, domain scores were also made by averaging the three total scores within each domain, and a final score by averaging all three domain scores. For the MT, a final score was calculated by averaging over the four games. Subsequently, we computed a mean training score for all three training groups separately and transformed these to Z-scores to be able to compare MT and experimental training groups relative to each other. The gain score was calculated by subtracting the mean score attained after the first 10 min of playing from the mean score attained at the end of training.

### Statistical analysis

A first set of repeated-measures ANOVAs focused on the executive functions in the principal analysis, using time points T0 (baseline) and T2 (post-training). Scores on task switching and dual tasking, updating, and inhibition were used as dependent variables, with group (FS, IS or MT) as the independent variable. A second set of repeated-measures ANOVAs was carried out for the secondary measures. PASAT, ToL, TMT-A, and TMT-B were transformed due to non-normality. PASAT scores were raised to the 3rd power, a square root transformation was used on ToL data, and TMT-A and TMT-B scores were transformed using the formula 1/x^0.14^. When necessary, outcome measures were rescored so that a positive value indicated improvement. We computed correlations between significant transfer tasks and age, TICS score, and workouts completed to determine whether to add them as covariates to the primary and secondary ANOVAs. Education level required non-parametric correlation analysis (Spearman's Rho); all other measures used Pearson's correlation coefficient. To explore the extent to which individual characteristics influenced training benefits, significantly correlated covariates were added to a repeated-measures ANCOVA of the primary and secondary measures.

When a significant improvement was detected at T2 on one of the dependent variables also measured at T1 (after 6 weeks of training) and T3 (post-training follow up after 4 weeks), these were additionally added to the model to establish whether training effects were visible after 6 weeks of training, and whether they remained after training had ceased.

Grubbs' Extreme Studentized Deviation test was used to detect outliers (Grubbs, 1950). We ran analyses with- and without outliers. All reported results are without outliers, unless otherwise specified. IBM SPSS Statistics for Windows, version 22 (IBM Corp., Armonk, N.Y., USA) was used for all statistical analyses. Normality was checked using Shapiro-Wilk's test and by evaluating skewness and kurtosis. A *p*-value of 0.05 (two-tailed if not mentioned otherwise) was considered significant. For all analyses, Bonferroni corrections for multiple testing were used. Greenhouse-Geisser corrected degrees of freedom were used whenever sphericity was violated, though for the purpose of legibility the original degrees of freedom are reported.

## Results

Of the 158 subjects we tested on baseline, 1 person was excluded before starting the training due to difficulty understanding the transfer tasks, 5 experienced substantial health problems, 3 reported lack of time, 5 did not enjoy the training, and another 5 experienced technical issues. There was no difference in gender, TICS score, education level, or training group between the final sample and dropouts (all *p*'s > 0.19) though there was a significant difference in age [sustainers *M* = 67.77, *SD* 5.0; dropouts *M* = 72.3, *SD* = 7.8, *t*_(20, 134)_ = −2,454, *p* = 0.023]. The subsequent results are based on the remaining 139 subjects (age 60–80, *M* = 67.8, 60.4% female, mean years of education 13.7).

Because participants receiving MRI scans were only assigned to either the frequent switch training or the MT, these groups contain a higher number of participants than the infrequent switch condition. Fifty-six subjects were allocated to the frequent switch training, 33 subjects to the infrequent switch training, and 50 subjects to the MT. Before training, the three training groups did not differ in gender, level of education, TICS score, age, or computer experience (all *p'*s > 0.26), as expected after minimization (see Table 1). There was also no difference in fatigue or depression (all *p's* > 0.48). Of the 14 participants whose fatigue scores exceeded the cut-off of 35, 5 were in the frequent switch condition, 5 in the active control and 4 in the infrequent switch condition. On the exit questionnaire, an equal number of people in each group reported having started new activities or training other than ours [χ2_(2, *N* = 139)_ = 0.561, *p* = 0.77].

**Table 1:** Subject demographics.

	**FS (*n* = 56)**	**IS (*n* = 33)**	**MT (*n* = 50)**	***p***
Age	67.8 ± 5.0	67.9 ± 5.4	67.6 ± 5.1	0.97
Gender (% female)	64.3	63.6	54	0.51^a^
Level of education	5.9 ± 0.9	5.8 ± 0.7	5.9 ± 0.9	0.76
TICS score	35.6 ± 2.5	35.7 ± 2.3	35.2 ± 1.8	0.51
Prior computer use	5.6 ± 0.8	5.4 ± 1.6	5.8 ± 0.8	0.11
CIS-F	20.0 ± 11.5	21.1 ± 11.5	18.1 ± 11.80	0.48
HADS-D	2.2 ± 2.6	2.5 ± 2.6	2.1 ± 2.1	0.73

*Values are Mean ± SD; p-values are based on ANOVA unless otherwise specified; Prior computer use based on seven-point scale (from 1 = less than once a month to 7 = more than 4 h a day); Level of education based on seven-point scale (from 1 = unfinished primary school to 7 = university); FS, frequent switching experimental group; IS, infrequent switching experimental group; MT, mock training group; TICS, Telephone Interview for Cognitive Status; CIS-F, Checklist Individual Strength–Fatigue subscale; HADS-D, Hospital Anxiety Depression Scale-Depression subscale*.

a *p-value based on χ2*.

### Intervention

Average number of completed training sessions was 57.1 (28.6 h) and this did not differ between training groups [*F*_(2, 135)_ = 0.438, *p* = 0.65]. All three groups improved equally on all training tasks, judging from the z-scores [*F*_(2, 135)_ = 0.192, *p* = 0.826], though in terms of total gain, the experimental conditions improved significantly more than did the active control [*F*_(2, 135)_ = 6.698, *p* = 0.002].

Although the active control condition was asked to maintain a single game level for a set week, we discovered that many active control participants continued playing beyond this level, thus diminishing differences in adaptiveness between our training groups. It appeared that 42% of control participants played more than 10% of their training time beyond the highest allowed level (level 9), and 26% played more than 30% of their time beyond this level. Besides this, 25 subjects (39% of IS subjects, 21% of FS subjects) scored a maximal number of points at the highest possible level on one or two of the nine games, thereby compromising adaptiveness among both experimental groups. Although many of these cases occurred only in the last few weeks of the training period, these events may have led to suboptimal differences between the MT and the experimental conditions.

Subjects were told after participation that we had made use of two conditions: one of which we expected would be less effective than the other. When asked whether they believed they had been in the more effective or less effective condition, participants were more likely to assume they had been in the experimental condition: 71% of FS and IS and 59% of MT expected they had received our more effective training. Neuropsychological assessors did not guess subjects' training group above chance level, both before training [39%; $\chi_{\left(4,\;\text{N}\;=\;105\right)}^{2}$ = 2.73, *p* = 0.60] and after training [33%; $\chi_{\left(4,\;\text{N}\;=\;105\right)}^{2}$ = 4.07, *p* = 0.39]. We can therefore assume that both neuropsychological assessors and participants themselves remained blind to the training conditions.

Besides this, there was no difference between the three conditions in the perceived difficulty or enjoyment of the games, self-rated cognitive enhancement, number of completed training sessions, or the degree to which they would like to continue playing the games (all *p*'s > 0.28), showing that all interventions were enjoyed equally.

### Transfer effects

The statistics of the primary and secondary analyses reported below are detailed in Table 2. Main effects of Group were absent throughout and will not be discussed further. ANCOVA outcomes are reported where appropriate.

**Table 2:** Transfer results.

**Group**								**Comparison**																
	**FS (*****n*** = **56)**				**IS(*****n*** = **33)**				**MT (*****n*** = **50)**				**Time**				**Group**				Time^*^Group			
**Measure**	**Pre-training**		**Post-training**		**Pre-training**		**Post-training**		**Pre-training**		**Post-training**		***df***	***F***	***P***	*$\eta_{P}^{2}$*	***df***	***F***	***P***	*$\eta_{P}^{2}$*	***df***	***F***	***P***	*$\eta_{P}^{2}$*
	***M***	***s.d***.	***M***	***s.d***.	***M***	***s.d***.	***M***	***s.d***.	***M***	***s.d***.	***M***	***s.d***.												
**PRINCIPAL**																								
Switch task	−348	175	−276	155	−368	266	−298	143	−367	225	−321	172	1;130	16.13	<**0.001**	0.11	2;130	0.238	0.79	0.004	2;130	0.535	0.59	0.01
Dual task	−1,215	274	−1,122	264	−1,241	302	−1,141	223	−1,290	300	−1,210	328	1;130	40.96	<**0.001**	0.24	2;130	0.702	0.5	0.01	2;130	0.078	0.93	0.001
SSRT	−258	48	−243	61	−269	56	−251	63	−265	61	−259	59	1;120	4.277	0.04	0.03	2;120	0.782	0.46	0.01	2;120	0.304	0.74	0.01
N-back	−10.2	7.4	−8.3	4.9	−9.2	4.5	−8.3	8.0	−11.4	7.8	−9.1	6.2	1;125	4.955	0.03	0.04	2;125	0.865	0.42	0.01	2;125	0.218	0.8	0.003
**SECONDARY**																								
D-KEFS TMT-4	−86.0	27.8	−80.7	31.0	−87.1	30.1	−71.1	22.1	−88	34	−77.8	31.7	1;133	16.796	<**0.001**	0.11	2;133	0.299	0.74	0.004	2;133	1.387	0.25	0.02
TMT-B	−71.7	29.3	−61.8	20.0	−72.6	31.7	−64.1	20.4	−71.3	21.0	−63.4	17.1	1;120	31.211	<**0.001**	0.21	2;120	0.312	0.73	0.01	2;120	0.519	0.6	0.01
Sem. fluency switch	−3.2	4.1	−3.6	4.2	−2.9	3.5	−5.3	4.5	−4.8	5.2	−4.8	4.1	1;129	3.653	0.06	0.03	2;129	2.362	0.1	0.04	2;129	1.66	0.19	0.03
Drag-and-drop	−37.3	9.7	−34.8	9.2	−39.2	8.5	−33.7	6.3	−42.6	17.1	−36.1	7.9	1;119	17.399	<**0.001**	0.13	2;119	1.877	0.16	0.03	2;119	1.289	0.28	0.02
Drag-to-grid	−62.8	13.5	−57.8	11.8	−66.4	13.1	−59.3	9.1	−65.3	12.2	−58.6	10.6	1;120	58.206	<**0.001**	0.33	2;120	0.535	0.59	0.01	2;120	0.706	0.5	0.01
Click task	−30.7	12.6	−27.3	8.2	−29.9	13.5	−26.0	6.0	−30.0	12.8	−25.2	7.1	1;118	12.549	**0.001**	0.1	2;118	0.333	0.72	0.01	2;118	0.159	0.85	0.003
TMT-A	40.6	11.3	37.4	7.0	42.9	10.4	36.7	8.6	43.9	15.2	37.4	7.7	1;119	27.518	<**0.001**	0.19	2;119	0.713	0.49	0.01	2;119	2.137	0.12	0.04
D-KEFS TMT-5	−28.1	9.6	−27.3	10.3	−26.2	7.9	−25.8	9.8	−27.8	9.5	−26.8	8.5	1;136	2.135	0.15	0.02	2;136	0.405	0.67	0.01	2;136	0.093	0.91	0.00
DSC	66.4	12.8	69.6	14.0	66.4	13.6	68.5	12.5	65.8	13.0	68.6	13.8	1;136	20.666	<**0.001**	0.13	2;136	0.054	0.95	0.001	2;136	0.255	0.78	0.00
DSC online	37.9	5.3	40.0	6.3	37.5	4.7	40.0	4.5	37.2	4.9	39.9	4.9	1;108	66.755	<**0.001**	0.38	2;108	0.082	0.92	0.002	2;108	0.498	0.61	0.01
ToL	33.5	23.3	27.1	17.9	33.4	21.4	22.4	15.9	37.0	24.4	22.3	19.1	1;123	24.059	<**0.001**	0.16	2;123	0.302	0.74	0.01	2;123	1.392	0.25	0.02
RPM	18.3	1.6	17.8	1.8	18.2	1.7	18.8	1.4	18.2	1.5	18.3	1.5	1;129	0.05	0.82	0	2;129	0.929	0.4	0.01	2;129	3.378	0.04	0.05
Shipley	15.3	2.8	15.9	3.0	15.8	2.7	17.1	2.5	15.8	2.7	16.5	2.7	1;130	23.496	<**0.001**	0.15	2;130	1.193	0.31	0.02	2;130	1.001	0.37	0.02
Corsi online	5.8	1.2	5.9	0.9	5.9	1.5	5.9	1.2	5.6	1.2	5.9	1.0	1;122	0.573	0.45	0.01	2;122	0.290	0.75	0.01	2;122	0.297	0.74	0.01
PASAT	96.7	16.5	103.5	16.2	96.6	14.6	103.6	11.2	93.8	16.5	101.2	13.5	1;133	67.008	<**0.001**	0.34	2;133	0.683	0.51	0.01	2;133	0.13	0.88	0.00
OSPAN	52.5	13.8	54.3	13.1	51.9	12.2	54.3	15.9	53.4	10.8	56.9	10.6	1;89	3.69	0.06	0.04	2;89	0.324	0.72	0.01	2;89	0.188	0.83	0.00
LNS	10.3	2.2	10.4	2.5	9.9	2.4	10.5	2.6	10.2	2.4	10.6	2.4	1;136	4.663	0.03	0.03	2;136	0.081	0.92	0.001	2;136	0.478	0.62	0.01
RAVLT	47.6	9.5	49.2	10.2	45.5	9.1	48.1	9.8	46.1	9.4	48.1	8.8	1;131	11.854	<**0.001**	0.08	2;131	0.435	0.65	0.01	2;131	0.219	0.8	0.00
RAVLT delay	10.6	3.1	11.1	3.0	9.3	2.9	10.0	3.2	10.4	2.8	10.6	2.8	1;131	5.744	0.02	0.04	2;131	1.853	0.16	0.03	2;131	0.599	0.55	0.01
Sem. fluency	23.4	4.8	23.4	5.7	22.6	4.6	24.1	5.3	23.8	5.1	23.2	5.2	1;129	0.487	0.49	0	2;129	0.014	0.99	0.001	2;129	2.146	0.12	0.03
Letter fluency	47.4	13.1	47.8	13.1	43.8	12.2	43.9	11.4	44.4	10.9	44.6	10.9	1;130	0.13	0.72	0	2;130	1.408	0.25	0.02	2;130	0.011	0.99	0.00

*p-values are based on repeated-measures ANOVA; Bonferroni-corrected significant values in bold; $\eta_{\text{p}}^{\text{2}}=$ partial eta squared (effect size); FS, frequent switching experimental group ; IS, infrequent switching experimental group; MT, mock training; SSRT, stop-signal reaction time; D-KEFS, Delis-Kaplan Executive Function System; TMT, Trail Making Test; sem, semantic; DSC, Digit Symbol Coding; ToL, Tower of London; RPM, Raven's Progressive Matrices; PASAT, Paced Auditory Serial Addition Test; LNS, Letter Number Sequencing; RAVLT, Rey Auditory Verbal Learning Test. TM T A and B, DKEFS 4 and 5, Drag-and-drop, Drag-to-grid and Click task in sec.; switchtask, dual task, SSRT in msec.; N-back in % correct*.

### Principle analysis: executive functions

On task switching, all three groups significantly improved their scores over Time, but a Time ^*^ Group interaction did not reach significance. A similar pattern was seen on the dual task, with a main effect of Time that was not modulated by Group. This time effect disappeared when correcting for the number of workouts [*F*_(1, 128)_ = 0.721, *p* = 0.397, $\eta_{p}^{2}$ = 0.006]. Time effects for N-back and Stop-signal task did not survive Bonferroni correction, and no modulation by Group was found. Equivalent results appeared when outliers were included.

### Secondary analysis

Most of the secondary measures were subject to improvement with Time, as described below, but none of these effects were modulated by Group. Performance on 2 out of 3 cognitive flexibility tasks improved over time for all three groups. Both the DKEFS TMT and the online version of the TMT-B showed decreased switching latency. No significant effects were found for performance on the switch condition of the semantic fluency task. Psychomotor speed improved on the online TMT-A and the three mouse ability tasks (Click, Drag-and-drop, and Drag-to-grid). The motor speed condition of the DKEFS TMT did not show a significant Time effect. Processing speed improved in both the original DSC as the online version of the task. A significant Time effect was found for the ToL. In the reasoning domain, a significant Time effect was observed for the SILS. The score on the RPM did not change significantly. On tasks of working memory, both PASAT and RAVLT-direct improved over Time, whereas no change appeared on the online Corsi or the Operation Span. The score on the LNS was not significant after Bonferroni correction. No Time effect was found on long term memory measured with the RAVLT-delay. Finally, both semantic and letter fluency did not improve over Time for any of the groups. With outliers included in the data, the results showed the same pattern.

All Time effects disappeared when correcting for age, baseline TICS score, number of completed training sessions, or education level, regardless of whether one or multiple covariates were used.

### Follow-up effects

As specific training effects were lacking, T1 measurements were not examined. An explorative repeated-measures ANOVA was run for tasks which did exhibit a significant Time effect and had also been administered at T3. All of these measures improved even further on T3, revealing higher effect sizes for all tasks [Switch task: *F*_(1, 125)_ = 59.167, *p* < 0.001, $\eta_{p}^{2}$ = 0.32; ToL: *F*_(1, 115)_ = 37.237, *p* < 0.001, $\eta_{p}^{2}$ = 0.25; online DSC: *F*_(1, 100)_ = 101.421, *p* < 0.001, $\eta_{p}^{2}$ = 0.50; TMT-A: *F*_(1, 112)_ = 43.755, *p* < 0.001, $\eta_{p}^{2}$ = 0.28; TMT-B: *F*_(1, 112)_ = 53.234, *p* < 0.001, $\eta_{p}^{2}$ = 0.32; Click task: *F*_(1, 112)_ = 16.933, *p* < 0.001, $\eta_{p}^{2}$ = 0.13; Drag-and-drop: *F*_(1, 109)_ = 39.465, *p* < 0.001, $\eta_{p}^{2}$ = 0.27; Drag-to-grid: *F*_(1, 113)_ = 60.085, *p* < 0.001, $\eta_{p}^{2}$ = 0.35]. However, these Time effects did not interact with Group for any of these measures, and no Time effects remained when correcting for age, education level, baseline TICS score, and number of completed training sessions, regardless of whether one or multiple covariates were used.

### Extra analyses

We added an extra analysis, examining a possible interaction between Time, Group, and switch task trial type (switch- and non-switch trials). This three-way interaction was not significant [F_(6, 360)_ = 0.233, *p* = 0.943, $\eta_{p}^{2}$ = 0.004].

To examine whether there was a significant difference in training benefit of Group after adjusting for baseline performance, we ran a separate number of ANCOVA's using difference scores on all measures, including baseline scores as a covariate. There was a difference in score on the Raven's Progressive Matrices [*F*_(2, 128)_ = 4.111, *p* < 0.019, $\eta_{p}^{2}$ = 0.060] between the frequent- and infrequent switch conditions, when adjusting for baseline RPM score. However, this value did not survive Bonferroni correction.

## Discussion

We investigated the possibility to train cognitive functioning in older adults using a computerized cognitive training. For this purpose, we designed an intervention with multimodal, novel, adaptive training tasks, a built-in element of flexibility, and frequent training sessions to optimize transfer, and selected a number of transfer tests with parallel forms to minimize retest effects. Based on previous literature (Mahncke et al., 2006; Karbach and Kray, 2009; Düzel et al., 2010) we expected far transfer to several executive functions. Improvement over time was found on training tasks as well as on multiple transfer tasks covering all domains. Our primary analyses showed that older adults benefited from training across the main domains of executive function (updating, shifting, inhibition; Miyake et al., 2000). Our secondary analyses partially confirmed these findings: improvements were seen in planning, reasoning, two out of three cognitive flexibility tests, two out of five working memory tests, and two out of three psychomotor speed tests; while no improvement was observed for IQ, long-term memory, and fluency. Improvements were further amplified 4 weeks after training completion.

Most importantly, however, the experimental training that capitalized on flexibility, novelty, and adaptiveness as central features did not lead to more progress than the trainings without these elements. This suggests that there was no additional advantage of these key ingredients in training tasks, and that improvements were induced mainly by other causes.

On outcome measures where a covariate was included, all time effects disappeared. Although covariates were added only if a significant correlation with a measure occurred, on plotting the covariate data it appeared that different values of each covariate affected the various measures differently. This suggests that the covariation effects were not systematic across covariates, and therefore did not add to the model to explain training effects.

Our study had a number of limitations, some of which it shares with similar studies in the literature.

### Generic factors: motivation, expectation, and placebo effects

The effects on training benefits from training-non-specific factors such as attention, motivation, expectancy, and placebo may have played a larger role than anticipated. Long-term intensive training interventions are accompanied by degrees of personal attention as well as motivation that in themselves may suffice to enhance cognitive performance. Moreover, such programs may induce an expectancy to improve, which has proven to generate powerful placebo effects across a wide range of domains and paradigms (Boot et al., 2013b; Dougherty et al., 2016; Foroughi et al., 2016).

Thus, one explanation also for the present set of findings is that of a placebo or subject-expectancy effect. In the information booklet that aspirant participants received at the beginning of the study, we informed them about our intention to investigate whether benefit from training was a possibility, and stated our hope to find positive effects on cognitive functioning. Although it also explained that we were not sure whether this would be the case, we might have inadvertently given subjects the notion that we expected benefit, thus leading them to put extra effort in post-training performance. The finding that a majority of our participants had assumed to be in the experimental condition, may support this notion. A similar pattern has been observed in Foroughi et al. (2016), in which subjects who had responded to a suggestive flyer displayed more improvement on cognitive functions, compared to a control group responding to a non-suggestive flyer. Elsewhere, we will report on how participants perceived their progress subjectively. If a subject-expectancy or placebo effect has indeed influenced the current results, these improvements might appear in their subjective reports, shedding more light on the question of overall time-based improvement.

A similar, but slightly different interpretation is that of the Hawthorne effect (Green and Bavelier, 2008), referring to subjects' tendency to perform better on tasks when they are working toward a common goal or when a need for attention is satisfied by participating in research. In our case, as all three conditions received a certain amount of social stimulation, this might have led to increased motivation to perform well on the post-training measurements.

### Potential limitations: challenge levels, group composition, and social cohesion

Based on previous studies using computerized training (Basak et al., 2008; van Muijden et al., 2012; Ballesteros et al., 2014; Kühn et al., 2014), we assumed that using the current set of nine games—with 20 levels each—would provide ample variation and challenge for 12 weeks. Nonetheless, some evidence suggests this challenge was not always met in our experiment. First, a considerable number of participants found themselves reaching the maximum score for at least one of the games within a number of weeks before the end of the training, diminishing adaptiveness in these groups. In addition, on the exit questionnaire, some participants in the frequent switch training commented that the training was too simple or repetitive, or specifically criticized adaptiveness, reflecting that learning in the first half of levels was too gradual and in the second half too steep; we did not answer to all personal needs for challenge. An improvement for future studies is to implement more variable and novel activities tailored to individual demands to further optimize performance increases. Yet, by and large, most participants experienced the tasks as aptly challenging, with the levels of variability and adaptiveness contributing to that experience.

Training in our active control group, on the other hand, might have been too challenging. We had meant to generate novelty only in the frequent- and in FS conditions, and assumed that by selecting only four, less multimodal, games in the active control condition, novelty would be minimal. However, for many participants (across groups) playing games seemed in itself to be a sufficiently novel activity to incur small cognitive effects. Many participants had not previously used a mouse in the relatively fast manner that was necessary in our games and computer tasks. The strong transfer effects to mouse ability tasks in all three groups supports this assumption. Another point to consider is that the limitation on adaptiveness in the control condition was compromised by the fact that many participants continued past the maximally allowed weekly game level, causing the control condition to be more challenging than intended. Thus, our control condition may have unintentionally targeted similar functions as in the experimental conditions. This is especially evident when comparing our design to those of other studies. Overall, many training studies that find more evident transfer than the current study have employed active control conditions that appear distinctly less active than the experimental conditions, spending fewer hours on assigned tasks and having markedly less interaction with the researchers. Some only use a passive control condition, or none at all. Our results stress the value of including an active control condition that receives equal attention and training time, yet creates no overlap in the engagement of functions.

Furthermore, for many of our subjects, participating in the training involved more than just playing the games and may have included aspects such as following a link in an email to get to the online test batteries, downloading a new browser, (later) starting up the correct browser, and navigating to the right page. Although all of our subjects used their computer regularly and knew about basic internet use, such actions beyond the training itself often exceeded those of their usual activities and, thus, may have constituted a type of unintended cognitive training.

However, results from our stroke sample, described elsewhere (van de Ven et al., 2017), suggest that this has had only a minor effect. In this sample, we investigated effects of the TAPASS training in recovering stroke patients, including a no-contact waiting list condition. This group showed equal improvements to the experimental intervention and active control, including mouse ability tasks. This suggests that playing games or increased use of a mouse could not have been the main factor behind the transfer effects that we found. Instead, the improvements in this study, appearing in all three training groups, are more likely to have been caused by retest effects. Almost all tests with parallel forms did not reach significance in any of the groups. Also, there was no indication that improvement was limited to specific cognitive processes, as transfer effects were not exclusive to specific domains. Testing frequency thus seems to be the most important factor underlying these time effects. Although we included parallel tests where available, future studies might benefit from using parallel tests only, to minimize these retest-effects. Furthermore, using the statistical analysis used at present, we have not fully been able to uncover further knowledge of the individual differences in training benefit. More thorough analyses are necessary to provide additional insight into the individual learning processes and contribute to future interventions.

Our design largely lacked social interaction with other participants, which might have provided additional stimulation (Ybarra et al., 2008; Charles and Carstensen, 2010). Also, although the focus for this project was on the effectiveness of the popular home-based training tasks, some recent evidence reveals that for non-impaired older adults, individual at-home training might not be as effective as group training (Kelly et al., 2014; Lampit et al., 2014) or training sessions provided in the lab (Basak et al., 2008; Lövdén et al., 2012; Ballesteros et al., 2014). Among reasons given are optimization of adherence and compliance, as well as providing motivation to master more difficult training tasks. Possibly, participants in these studies may have benefited more from the training due to this procedure, producing more conspicuous results than the home-based training method presented here. Yet, as we contacted participants frequently with motivational telephone calls, it is unclear whether increased transfer in the experimental conditions would have occurred, if subjects had received face-to-face support from a trainer instead.

In our current study, we used a set of commercially available games targeted at the general population to train their cognitive functions. Naturally, commercial- and scientific games are created with different intentions in mind, yet in this case, we expect this to be less of a concern, as we took care to adapt each game, as well as the design of the intervention, to fit our scientific objectives, with the additional benefit of generalizing more to other functions than the frequently used commercial games.

A potential methodological limitation of this study was the homogeneity of our elderly sample, with a high educational level and relatively few cognitive complaints. This is characteristic of participants interested in volunteering in research experiments, increased further by the inevitable self-selection due to our inclusion criteria, such as the requirement to own a modern computer and to be willing to spend 12 weeks on our training. This raises the question to what degree cognitive improvement could have been attained in a sample with ample daily cognitive stimulation and minimal need to improve functions. A sample of older, less fit individuals might be more representative in displaying the benefit for the population. However, logistically it is difficult to encourage lower-educated, more cognitively impaired individuals to participate in research, let alone spend a sufficient amount of time on such an intervention. Despite subjects' demographic homogeneity, we noticed a large test score variability within groups, overshadowing any differences between them.

## Conclusion

Our cognitive flexibility training, using elements based on previously effective cognitive interventions, did not produce the expected near- and far transfer. Although training benefits were observed almost across the board, equal effects appeared in the active control group. Taken at face value, our results with commercially available training games suggest that this type of training may yield cognitive benefits among older adults. In our experimental design, however, we could not disentangle training effects from those attributable to test practice, expectancy, and motivation. Our parallel study with recovering stroke patients (van de Ven et al., 2017), which included a wait list condition, suggests that such factors may well have overshadowed the beneficial effect of the training itself and that training effects on cognition could be rather small. Additional investigation into different training methods is advised, including stimulation of social interaction and the use of more variable, novel, group-based yet individual-adjusted activities. Our results further emphasize the importance of using parallel forms as outcome measures for transfer and including both passive and active control conditions.

As a future direction, we may observe that a thus far underexplored territory pertains to individual differences in “trainability” or the susceptibility to benefits from particular aspects of a training. For instance, it may prove fruitful to explore which cognitive or neural connectivity profiles are predictive of who will improve in what domain. If brain training is to be successful in a meaningful way, we first have to learn more about which determinants are key in tailor-made interventions to maximize far transfer.

## Ethics statement

This study was carried out in accordance with the recommendations of the guidelines formulated by the Ethics Review Board of the Faculty of Social and Behavioral Sciences, University of Amsterdam, The Netherlands, with written informed consent from all subjects. All subjects gave written informed consent in accordance with the Declaration of Helsinki. The protocol was approved by the aforementioned Ethics Review Board of the University of Amsterdam.

## Author contributions

JB and RvdV contributed to conception and design of the study and were responsible for data collection. JB and SP analyzed the data. JB interpreted the data and wrote the manuscript. JB, RvdV, SP, JM, and KR critically revised the article and approved this version to be published.

### Conflict of interest statement

The authors declare that the research was conducted in the absence of any commercial or financial relationships that could be construed as a potential conflict of interest.
