# Supplementary material for: Cognitive Flexibility Training: A Large-Scale Multimodal Adaptive Active-Control Intervention Study in Healthy Older Adults
Source: Front Hum Neurosci. 2017 Nov 1;11:529. doi: 10.3389/fnhum.2017.00529 (PMC5701641; doi:10.3389/fnhum.2017.00529)
Supplement: Supplementary file 1 [file Image1.PDF]

Supplementary Material

Cognitive flexibility training: A large-scale multimodal adaptive active-control intervention study in healthy older adults.

Jessika I.V. Buitenweg*, Renate M. van de Ven, Sam Prinssen, Jaap M.J. Murre, K. Richard Ridderinkhof

*** Correspondence:** Jessika Buitenweg: j.i.v.buitenweg@uva.nl

1 Rating of performance during task

In all groups, participants acquire gold stars on screen for their performance on each game. An overview of which stars have been obtained per level can be seen at the beginning and end of each game.

For the FS and IS groups, if subjects obtain zero or one star they are asked to maintain training on the same level of difficulty. If two stars are achieved, they are encouraged to select the next level, but also allowed to stay on the same level to try to obtain three golden stars. If three stars are acquired, they are asked to pick the higher level of difficulty.

The MT group was asked to always select the same level during training, and only continue to a new level at the beginning of the training week. In weeks 6 and 7, and in weeks 9 and 10, the same level was played for two weeks.

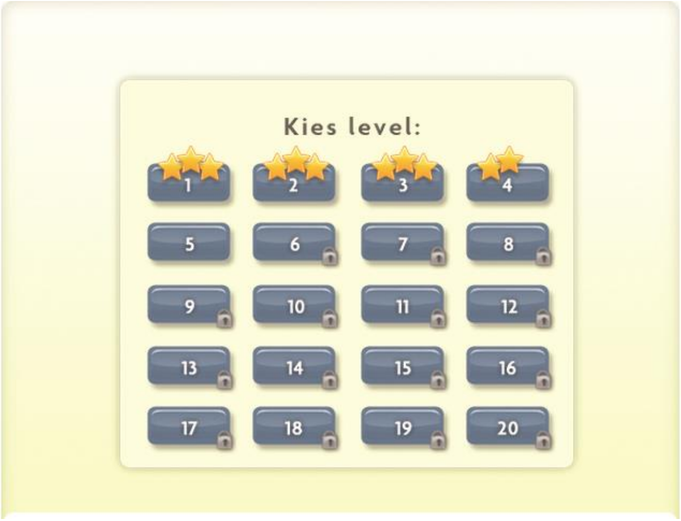

Supplementary Figure 1. Performance rating per game visible to participants.

2 Cognitive Training games

2.1 Memory training

Toy Shop: A shopping list with pictures of items is shown for a limited amount of time. Participants need to recall the items to collect them from a shelf.

Multi Memory: Cards are shown for a limited time, with figures in different colors and shapes. Participants have to reconstruct these figures after they have disappeared.

Moving Memory: Comparable to the well-known game of Memory, participants need to find pairs of cards. In this variation, the remaining cards change position after a pair has been found. Figures on the cards can only be remembered based on the number printed on the back, but no longer by the location.

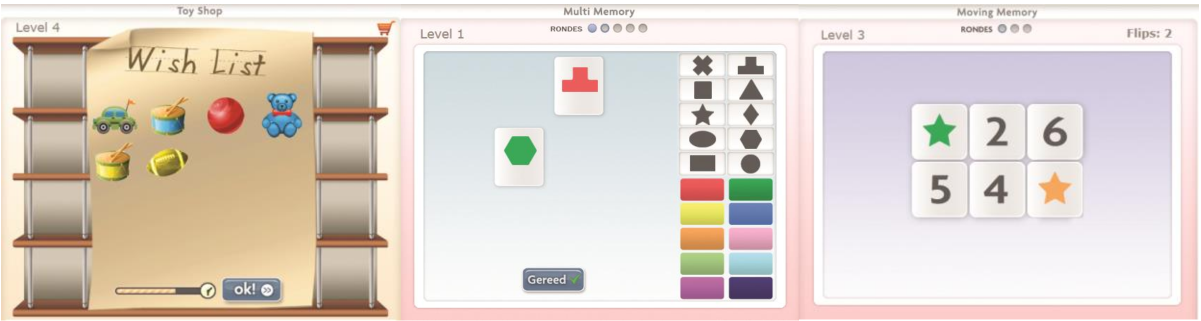

Supplementary Figure 2. Memory training games: Toy Shop, Multi Memory and Moving memory

2.2 Attention training

Mind the Mole: Molehills appear on screen one by one, each moving in a particular direction. Participants need to be vigilant for all molehills on screen and select one once it begins to move in a different direction before the mole takes away all the carrots.

Birds of a Feather: Under time pressure, participants have to count birds of a particular color and shape among other different birds.

Pattern Matrix: A number of tiles with different patterns on it are displayed. Participants need to mentally rotate the tiles to find all matching pairs within the given time.

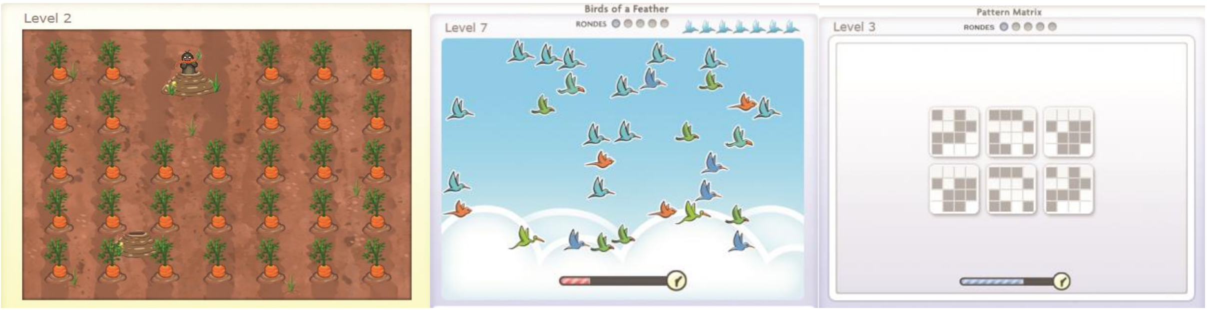

Supplementary Figure 3. Attention training games: Mind the Mole, Birds of a feather and Pattern Matrix

2.3 Reasoning training

Pattern Logic: A number of cards with figures on it are displayed in unknown serial patterns of color and shapes. Participants have to find the patterns and complete the missing tiles.

Out of Order: A row of tiles with figures in different shapes and colors need to be arranged under time pressure in such a way that they match with adjacent tiles on minimally one characteristic (e.g. color, shape, pattern or number of figures).

Square Logic: Cards containing a number have to be dragged on top of each other within the time given, such that only one block will remain. Cards can only be placed on adjacent cards with a number that is a count one higher or lower.

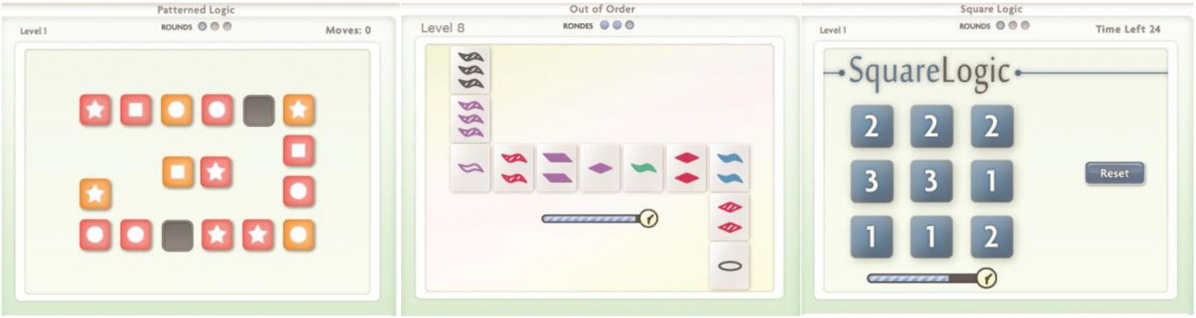

Supplementary Figure 4. Reasoning training games: Pattern Logic, Out of order and Square Logic

3 Active Control games

Pay Attention: Colored squares will appear randomly on the screen. Whenever the color of the square is changed, the participant has to select it as fast as possible.

Sliding Search: Pictures will slide from left to right over the lower part of the screen. Participants need to match pictures from the upper part with those in the lower part of the screen.

Grid Tracks: Participants have to mentally follow the trajectory of a number of tiles previously marked with a star. When the blocks stop moving, participants need to locate the stars from memory.

Fuzzle: Participants have to reconstruct a fractured picture within the given time.

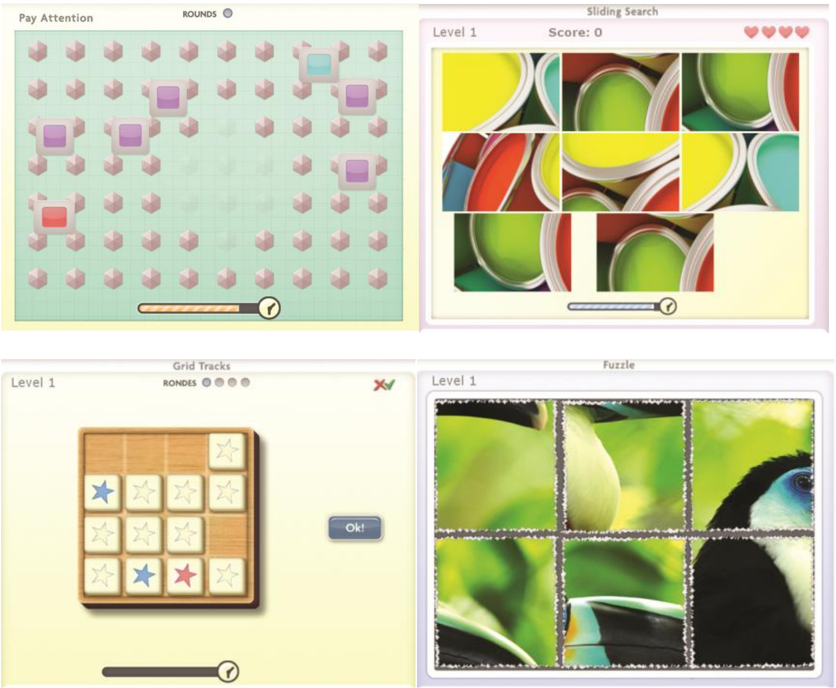

Supplementary Figure 5. Active control games: Pay Attention, Sliding Search, Grid Tracks, and Fuzzle
